# Supplementary material for: Structure-based design of an immunogenic, conformationally stabilized FimH antigen for a urinary tract infection vaccine
Source: PLoS Pathog. 2025 Feb 19;21(2):e1012325. doi: 10.1371/journal.ppat.1012325 (PMC12136410; doi:10.1371/journal.ppat.1012325)
Supplement: S1 Fig — (PDF) [file ppat.1012325.s001.pdf]

Fig. A.

A

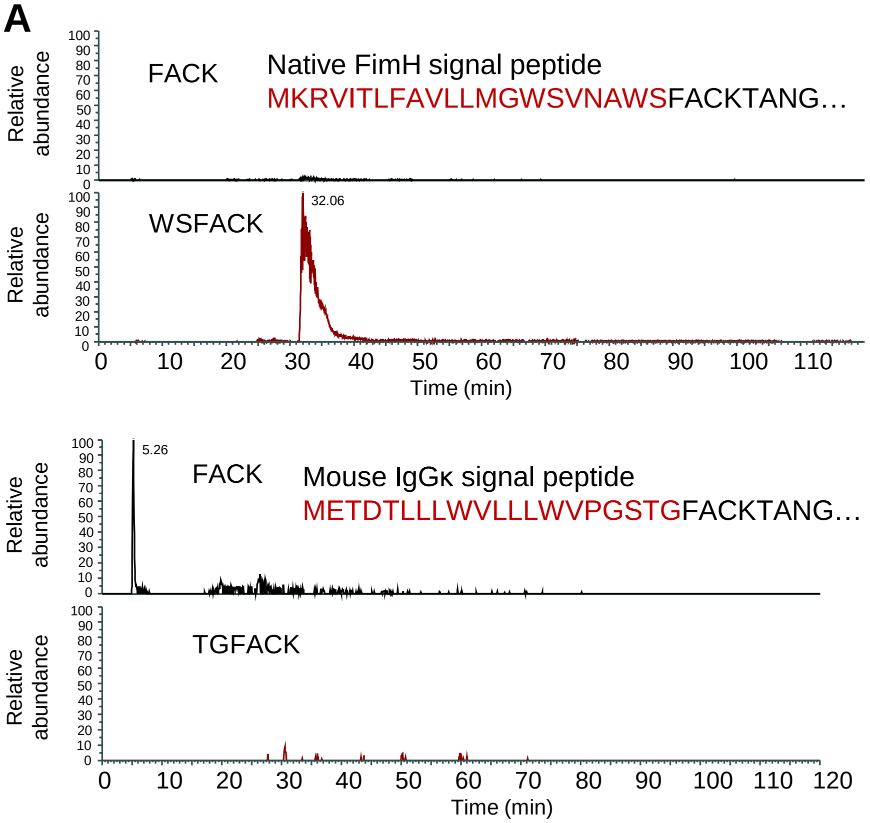

B

Parent Ion: 525.25 amu		
**b ion**	**Residue**	**y' ion**
148.1	F	-
219.1	A	378.2
379.1	C	307.1
-	K	147.1

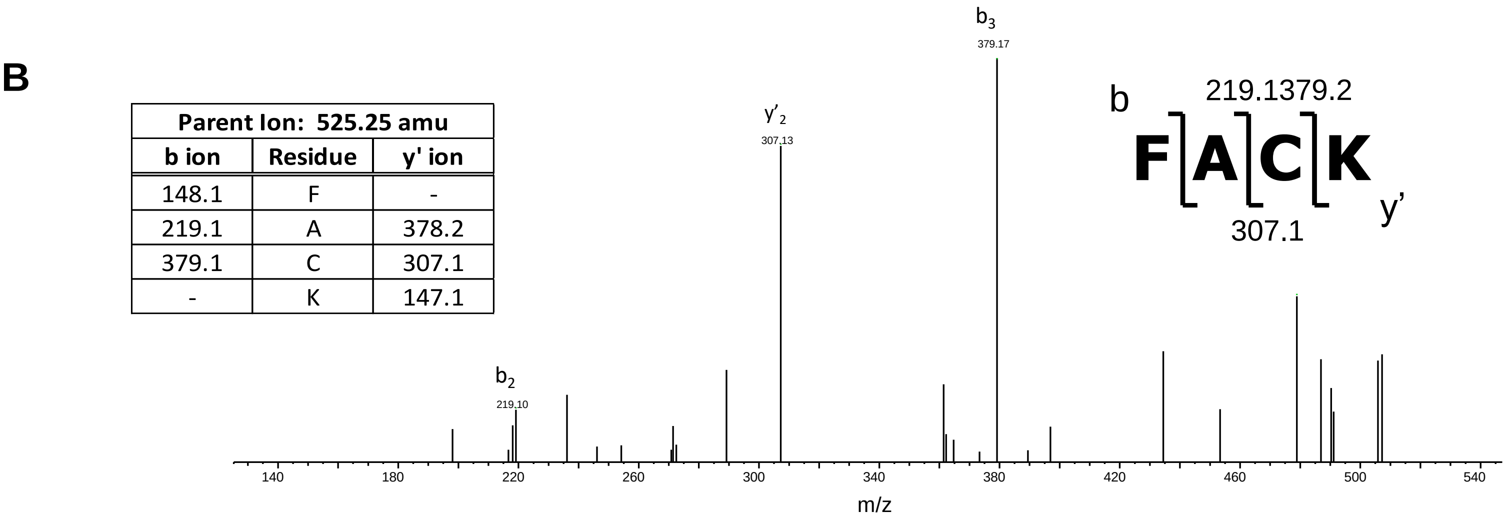

C

Parent Ion: 798.36 amu		
**b ion**	**Residue**	**y' ion**
187.1	W	-
274.1	S	612.3
421.2	F	525.2
492.2	A	378.2
652.3	C	307.1
-	K	147.1

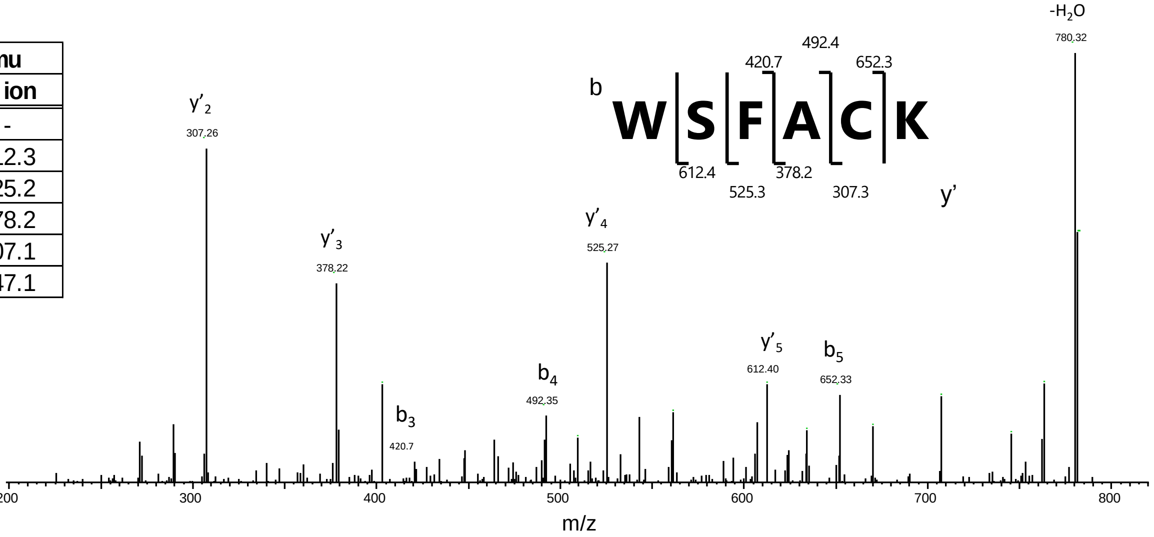

Fig. B.

A

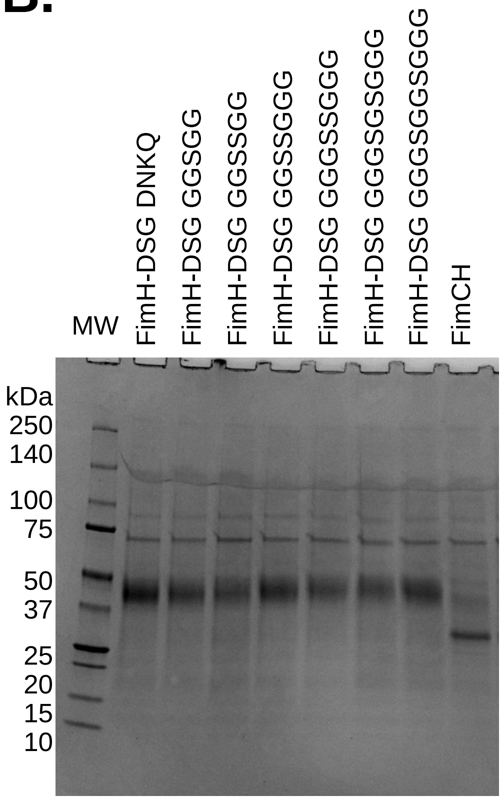
MW
FimH-DSG DNKQ
FimH-DSG GGSGG
FimH-DSG GGSSGG
FimH-DSG GGSSGGG
FimH-DSG GGGSSGGG
FimH-DSG GGGSGSGGG
FimH-DSG GGGSGGSGGG
FimCH
kDa
250
140
100
75
50
37
25
20
15
10

B

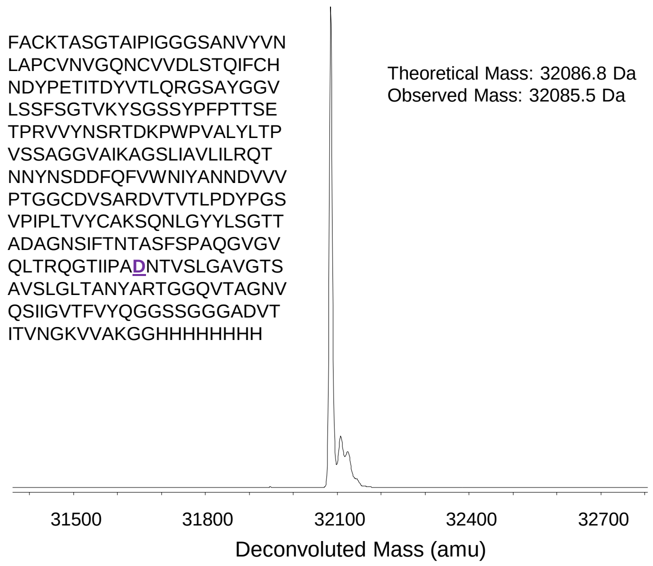
FACKTASGTAIPIGGGSANVYVN
LAPCVNVGQNCVVDLSTQIFCH
NDYPETITDYVTLQRGSAYGGV
LSSFSGTVKYSGSSYPFPTTSE
TPRVVYNSRTDKPWPVALYLTP
VSSAGGVAIKAGSLIAVLILRQT
NNYNSDDFQFVWNIYANNDVVV
PTGGCDVSARDVTVTLPDYPGS
VPIPLTVYCAKSQNLGYYLSGTT
ADAGNSIFTNTASFSPAQGVGV
QLTRQGTIIPADNTVSLGAVGTS
AVSLGLTANYARTGGQVTAGNV
QSIIGVTFVYQGGSSGGGADVT
ITVNGKVVAKGGHHHHHHHH
Theoretical Mass: 32086.8 Da
Observed Mass: 32085.5 Da
31500
31800
32100
32400
32700
Deconvoluted Mass (amu)

C

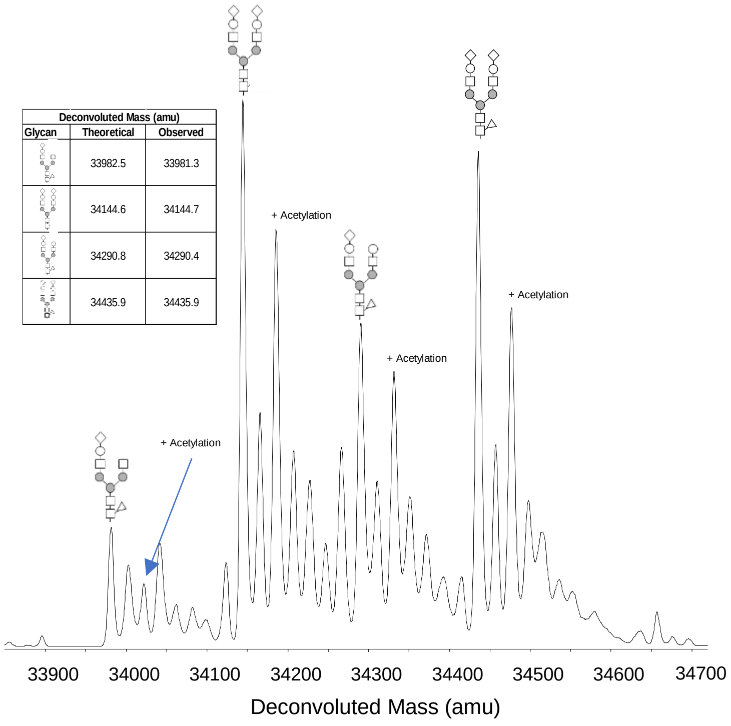
Deconvoluted Mass (amu)
Glycan
Theoretical
Observed
33982.5
33981.3
34144.6
34144.7
34290.8
34290.4
34435.9
34435.9
+ Acetylation
+ Acetylation
+ Acetylation
+ Acetylation
33900
34000
34100
34200
34300
34400
34500
34600
34700
Deconvoluted Mass (amu)

Fig. C.

A

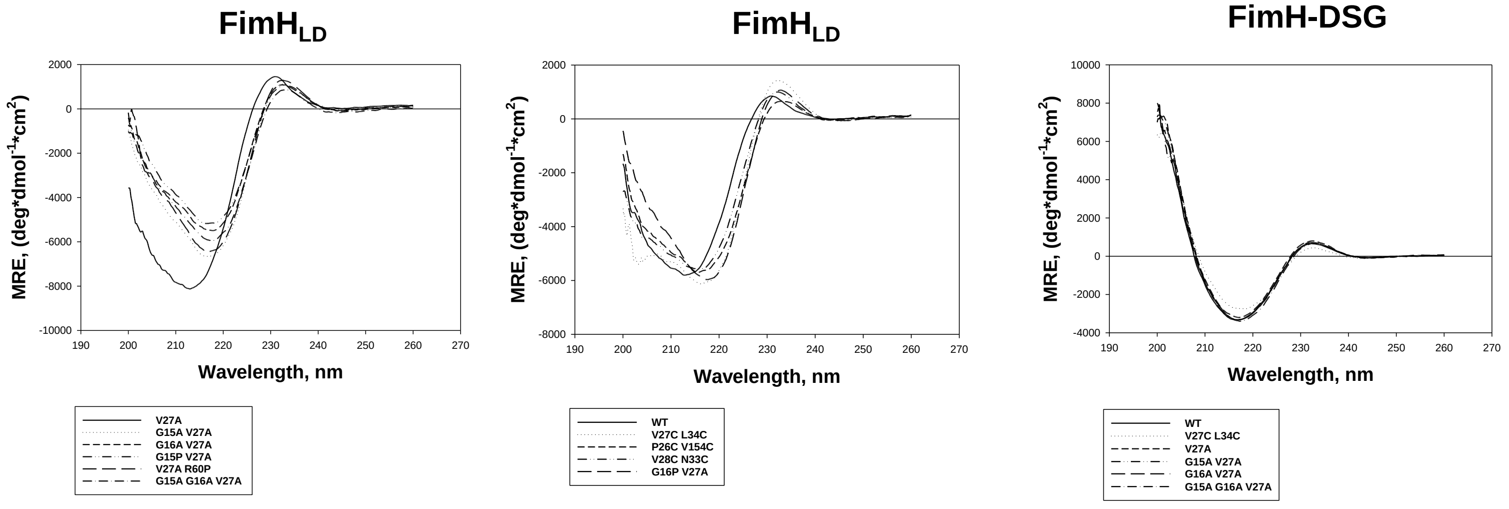
FimHLD
MRE, (deg*dmol-1*cm2)
Wavelength, nm
V27A
G15A V27A
G16A V27A
G15P V27A
V27A R60P
G15A G16A V27A
FimHLD
MRE, (deg*dmol-1*cm2)
Wavelength, nm
WT
V27C L34C
P26C V154C
V28C N33C
G16P V27A
FimH-DSG
MRE, (deg*dmol-1*cm2)
Wavelength, nm
WT
V27C L34C
V27A
G15A V27A
G16A V27A
G15A G16A V27A

B

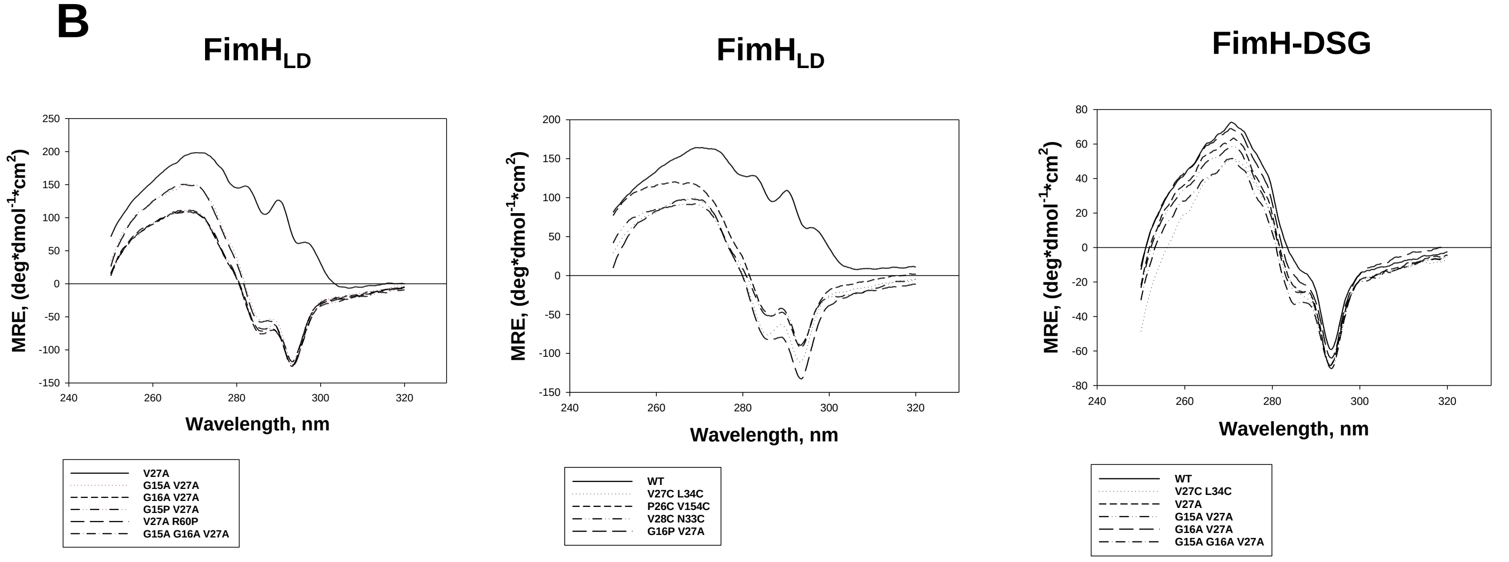
FimHLD
MRE, (deg*dmol-1*cm2)
Wavelength, nm
V27A
G15A V27A
G16A V27A
G15P V27A
V27A R60P
G15A G16A V27A
FimHLD
MRE, (deg*dmol-1*cm2)
Wavelength, nm
WT
V27C L34C
P26C V154C
V28C N33C
G16P V27A
FimH-DSG
MRE, (deg*dmol-1*cm2)
Wavelength, nm
WT
V27C L34C
V27A
G15A V27A
G16A V27A
G15A G16A V27A

Fig. D.

A

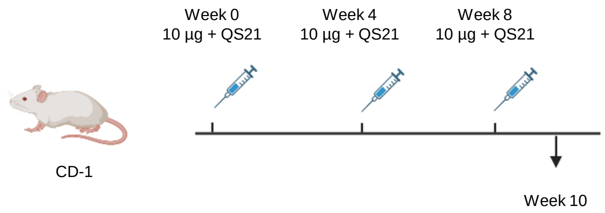

B

Group	Responder rate (%)	# of responders / N mice	Binding inhibition / geometric mean IC_{50}
FimH-DSG TM	89	16/18	525
FimH-DSG TM N235S	95	18/19	736
FimH-DSG TM N235Q	81	13/16	393

C

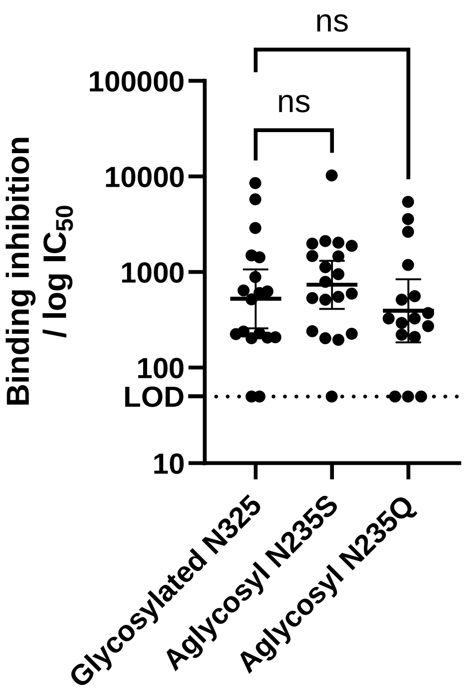

Fig. E.

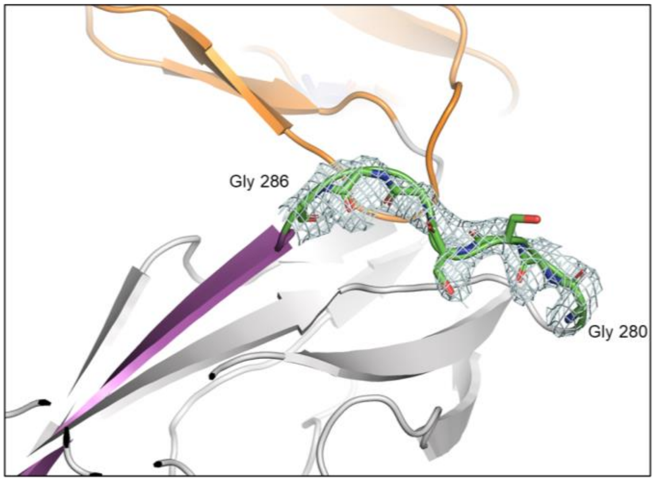

Fig. F.

FimH-DSG WT | FimH-DSG TM

A

SP-Sepharose

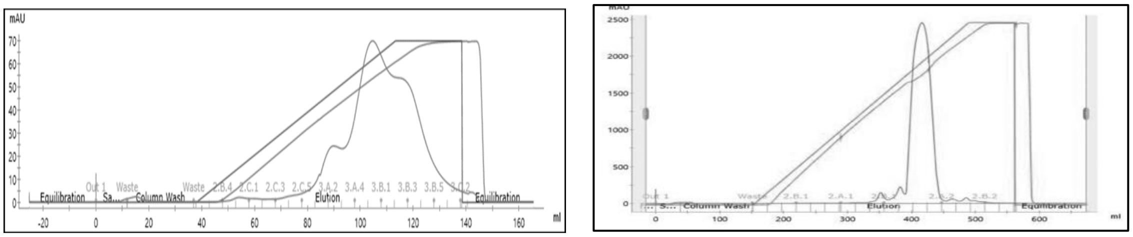

B

SDS-PAGE

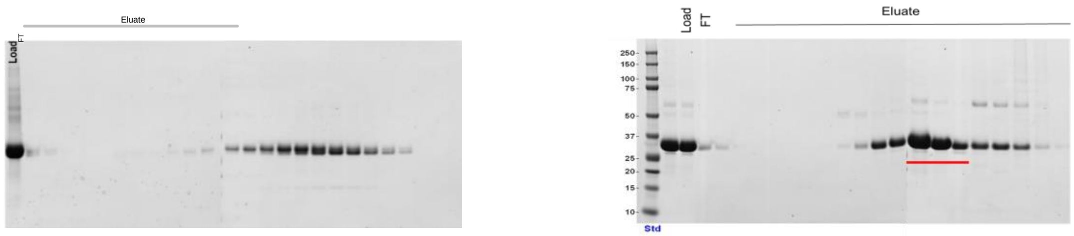

C

SEC

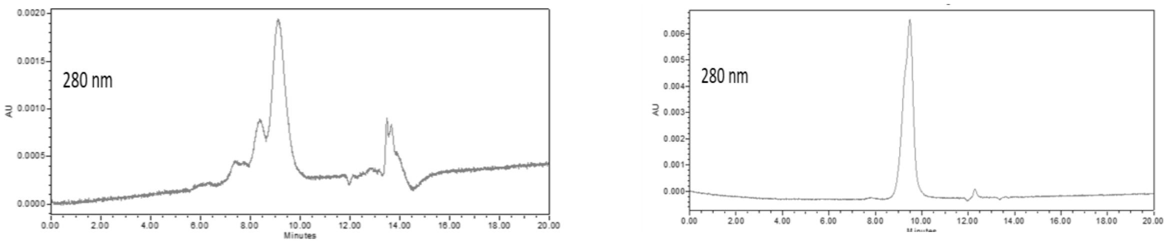

D

HPAEC-PAD

Sugar	Fraction 1	2	3	4	5	6	7	8	9	Total
Rhamnose	0	0	0	0	0	0	0	0	0	0
NAc Gal	0	0	0	99	73	0	52	0	177	401
NAc Glu	133	190	9	0	0	0	0	44	0	376
Galactose	24	21	6	0	0	0	41	33	35	160
Glucose	8	3	0	19	25	62	34	52	33	236
Mannose	47	35	10	0	0	0	34	59	56	241

Sugar	Fraction 1	2	3	4	5	6	7	8	9	Total
NAc Glu			20							20
Galactose			7							7
Mannose			17							17

Figure G.

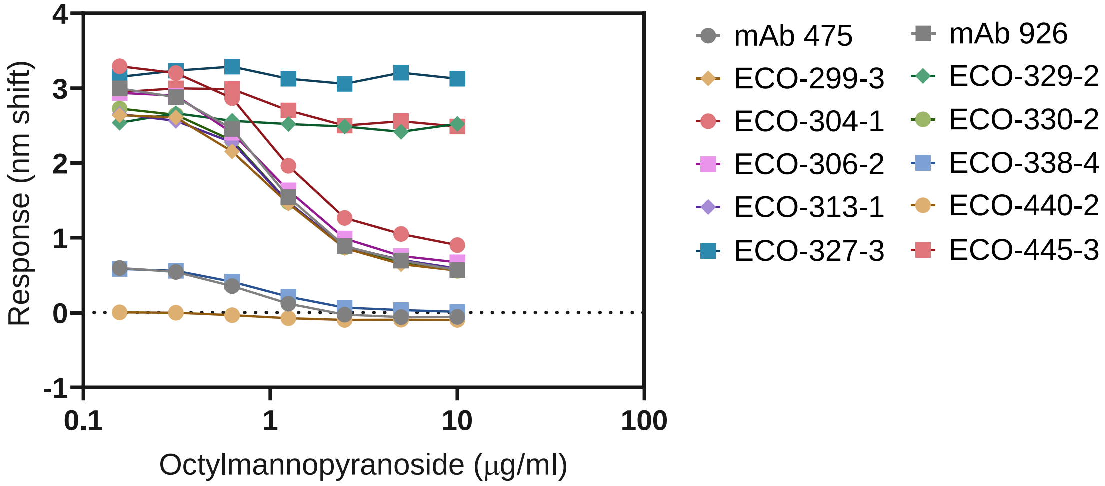

4
3
2
1
0
-1
Response (nm shift)
0.1
1
10
100
Octylmannopyranoside (µg/ml)
mAb 475
ECO-299-3
ECO-304-1
ECO-306-2
ECO-313-1
ECO-327-3
mAb 926
ECO-329-2
ECO-330-2
ECO-338-4
ECO-440-2
ECO-445-3

Fig. H.

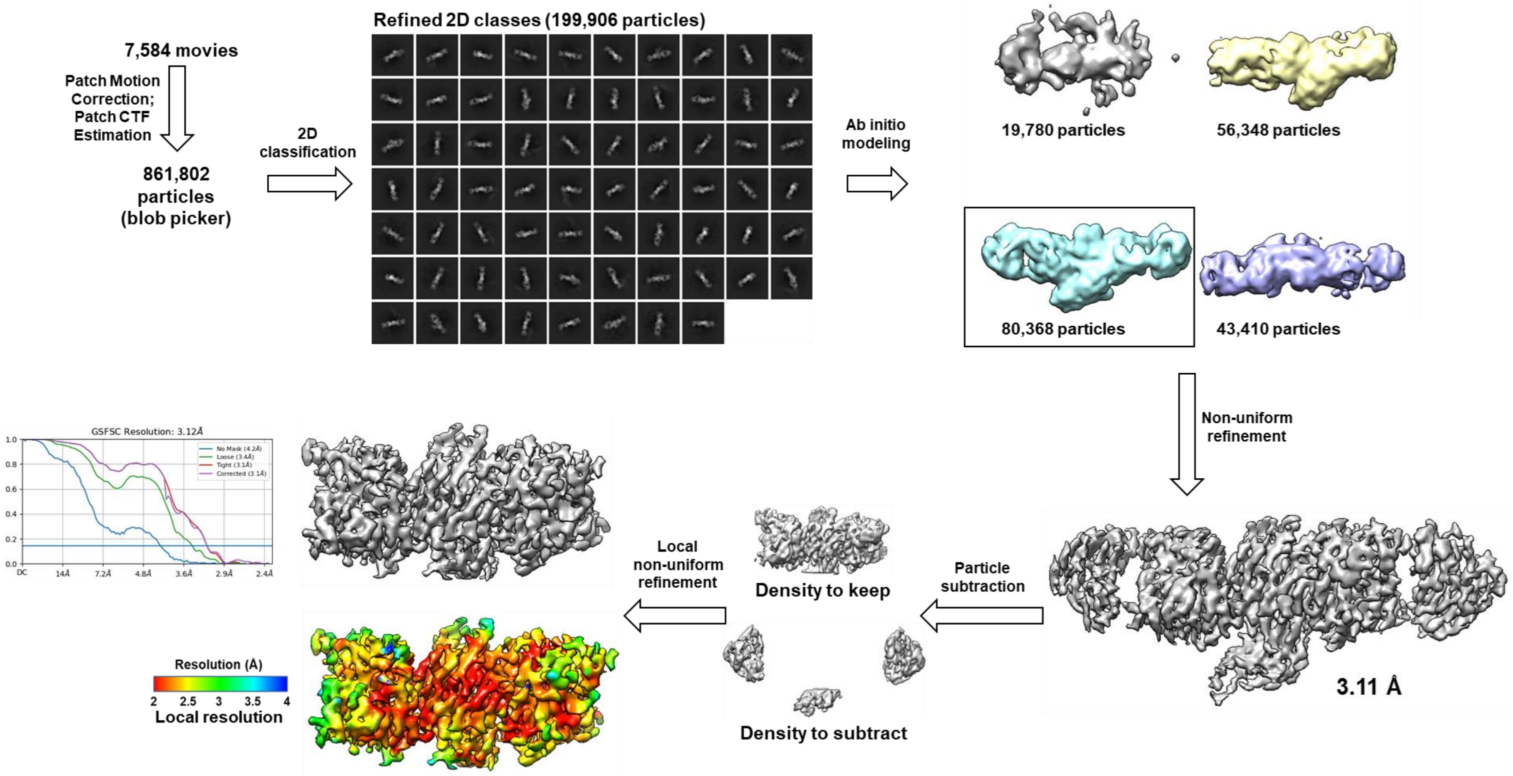
7,584 movies
Patch Motion Correction; Patch CTF Estimation
861,802 particles (blob picker)
2D classification
Refined 2D classes (199,906 particles)
Ab initio modeling
19,780 particles
56,348 particles
80,368 particles
43,410 particles
Non-uniform refinement
3.11 Å
Particle subtraction
Density to keep
Density to subtract
Local non-uniform refinement
GSFSC Resolution: 3.12Å
Resolution (Å)
2 2.5 3 3.5 4
Local resolution

Fig. I.

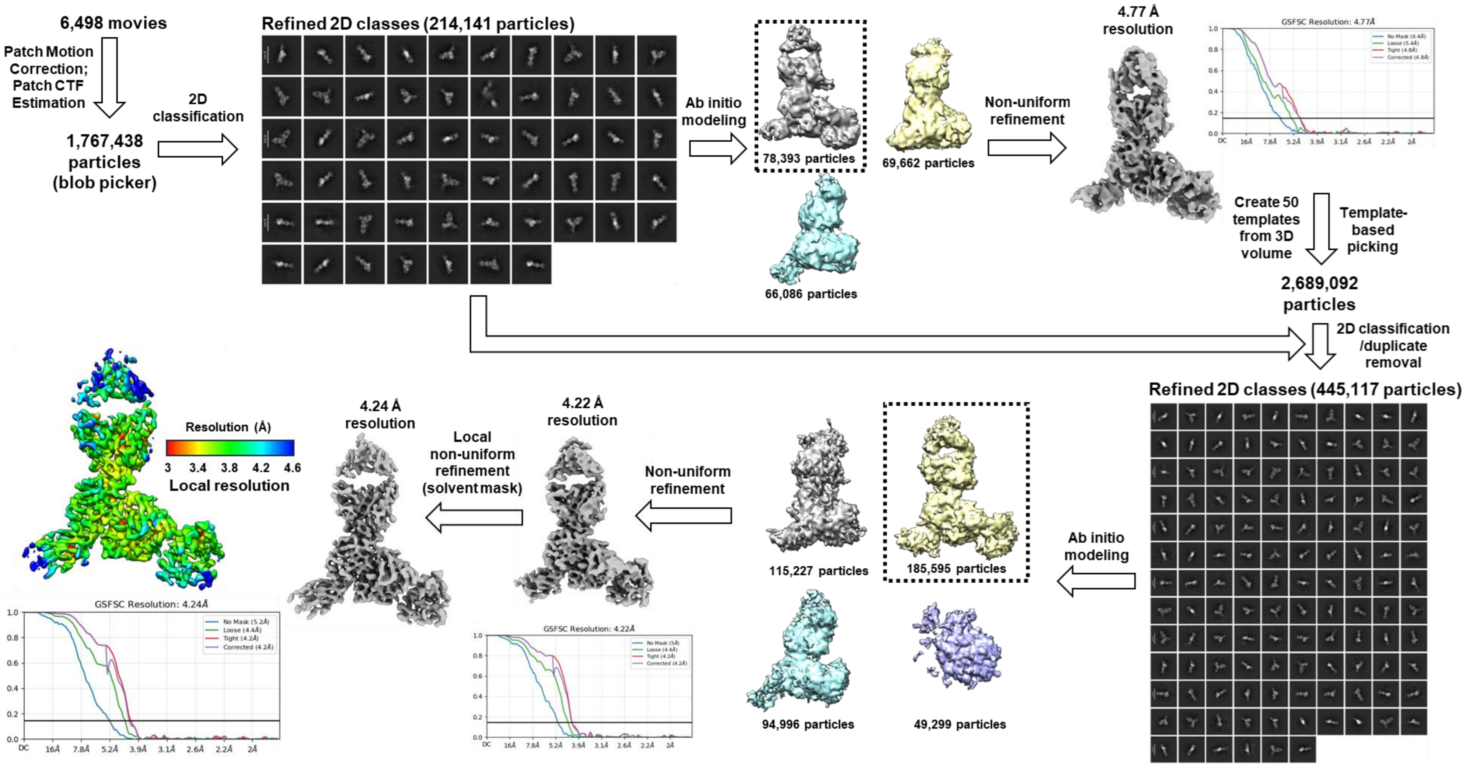

6,498 movies
Patch Motion Correction; Patch CTF Estimation
1,767,438 particles (blob picker)
2D classification
Refined 2D classes (214,141 particles)
Ab initio modeling
78,393 particles
69,662 particles
66,086 particles
Non-uniform refinement
4.77 Å resolution
GSFSC Resolution: 4.77Å
Create 50 templates from 3D volume
Template-based picking
2,689,092 particles
2D classification /duplicate removal
Refined 2D classes (445,117 particles)
Ab initio modeling
115,227 particles
185,595 particles
94,996 particles
49,299 particles
Non-uniform refinement
4.22 Å resolution
GSFSC Resolution: 4.22Å
Local non-uniform refinement (solvent mask)
4.24 Å resolution
GSFSC Resolution: 4.24Å
Resolution (Å)
3 3.4 3.8 4.2 4.6
Local resolution
