# Supplementary material for: Structure-based design of an immunogenic, conformationally stabilized FimH antigen for a urinary tract infection vaccine
Source: PLoS Pathog. 2025 Feb 19;21(2):e1012325. doi: 10.1371/journal.ppat.1012325 (PMC12136410; doi:10.1371/journal.ppat.1012325)
Supplement: S1 Text — (DOCX) [file ppat.1012325.s003.docx]

**Supporting information**

**Table of Contents**

[1. Supplementary Methods 3](#_Toc185858585)

[**1.1.** **Bioinformatics analysis of FimH sequences** 3](#_Toc185858586)

[**1.2.** **Characterization of FimH proteins** 3](#_Toc185858587)

[**1.3.** **X-ray crystallography of FimH-DSG TM** 6](#_Toc185858588)

[**1.4.** **Binning of monoclonal antibodies** 7](#_Toc185858589)

[**1.5.** **Sample Preparation for FimH-DSG TM Fab complex Cryo-EM** 8](#_Toc185858590)

[**1.6.** **Cryo-EM Data Collection and Processing** 8](#_Toc185858591)

[**1.7.** **Model Building and Refinement** 10](#_Toc185858592)

[2. Supplementary data tables 12](#_Toc185858593)

[3. Supplementary figures 28](#_Toc185858594)

[Figures: can be found in Silmon de Monerri et al supplementary figures.pdf 28](#_Toc185858595)

[Figure legends 28](#_Toc185858596)

1. **Supplementary Methods**
   1. **Bioinformatics analysis of FimH sequences**

Amino acid sequences of FimH mutants evaluated and wild type FimH from *E. coli* strain J96 (from NCBI, GCA_000295775.2) were assembled in Geneious and aligned using the default Geneious algorithm. The J96 sequence was set to reference. 102 FimH amino acid sequences from whole genome sequencing data of contemporary UTI isolates (1) were assembled in Geneious and aligned with FimH from J96 and FimH-DSG TM using the default Geneious algorithm.

- 1. **Characterization of FimH proteins**

*Mass spectrometry*

Intact mass analysis was carried out on an Agilent 6530 Qtof Mass spectrometer coupled with an Agilent Infinity 1290 UPLC chromatography system using electrospray ionization with HP-621 as a reference mass (hexakis(2,2 difluoroethoxy)phosphazene). The proteins were separated on an PLRP-S 1000A, 2.1 X 50 mm, 5u column (p/n: PL1912-1502) using a linear gradient of water and acetonitrile with 0.1% formic acid starting at 95% water. Resulting spectra were deconvoluted using MassHunter v7.0 with BioConfirm v8.0.

Determination of the processing of the N-terminus was investigated by enzymatic digestion of the proteins and subsequent LC-MS/MS analysis for confirmation. An aliquot of the protein was precipitated with ice cold acetone and incubated overnight at -20°C. The protein was pelleted, dried and resuspended in 8M urea with 20 mM methylamine. The sample was reduced with DTT and alkylated with iodoacetamide using standard protocols. The resulting sample was digested with trypsin (100 mM Tris-HCl, 10 mM CaCl2; 1:20 enzyme:protein ratio by weight) at 37°C overnight.

The resulting peptides were then introduced to a Thermo LTQ XL Orbitrap mass spectrometer outfitted with an Aligent 1100 capillary HPLC to perform separations. The system was operated in direct injection mode and the peptides were chromatographically separated using a Higgins PROTO 300 C18 100X0.5mm, 5mm, column (p/n RS-10M5-W185) with a flow rate of 10 mL/minute. The mass spectrometer was run in data dependent mode using a top 3 ion method. Dynamic exclusion was enabled with a repeat count of 2 and an exclusion duration of 30 seconds. Peptides were fragmented using a normalized collision energy of 35 with a default charge state of 2. The isolation width was set to 3 Da. The full MS scan resolution was set to 15000 and MS/MS was collected in CID in the ion trap. Data analysis was enabled using Matrix Sciences MASCOT database search engine where unique protein sequences can be added to an In-House database.

*Fluorescence polarization assay.* To determine dissociation constants of FimH mutants for mannoside ligands, a fluorescence polarization assay was developed based on methods described by Rabbani et al (2) [u](#_ENREF_17)sing fluorescein conjugated to mannoside ligands with a high affinity for FimH. FimH proteins were diluted in 20 mM HEPES pH 7.4, 150 mM NaCl, 0.05 mg / ml plus BSA 0.05% in an 11-point threefold titration in a black flat bottom 96-well polypropylene plate (Greiner) with a final volume of 50 µl. 50 µl of fluorescein octylbiphenylmannopyranoside ligand at 0.7 nM in same buffer was added to each well. Plates were incubated overnight at room temperature, shaking at 100 rpm. After 20-24 hours, plates were read in a ClarioStar Plus plate reader with fluorescein excitation at 488 nm and emission at 530 nm.

*Circular dichroism (CD) spectroscopy*

Far-UV (320-250 nm) and near-UV (260-200 nm) circular dichroism spectra were recorded for FimH_LD_ and FimH-DSG mutants using JASCO J-810 Spectropolaromiter (Jasco), equipped with JASCO PTC-424S/15 (Jasco) temperature control and Isotemp water bath (Fisher Scientific) units. For far-UV, a 1 mm cell was used, and for near-UV a 10 mm cell was used. Proteins were diluted to 0.3 mg / mL in PBS and spectra were recorded at 20 ºC using a cell with 1 mm (far-UV) or 10 mm (near-UV) path length. Scans were performed at 100 nm / min, DIT was set to 1s, bandwidth to 3s, and data pitch to 0.1 nm. Sensitivity was set to standard. Ten spectra were accumulated and averaged for near-UV and five for far-UV measurements respectively. Spectra were corrected to background manually using CD spectra arising from blank PBS runs and were converted to mean residue ellipticity using EQ. 1. Where 𝚹MRE is the calculated mean residue ellipticity, 𝚹EXP is experimentally measured CD signal, MW is protein molecular weight, N is the number of amino acid residues, C is protein concentration in mg/mL, l is the optical path length in cm.

EQ. 1ϴ_MRE_=(ϴ_EXP_•MW)/(10•N•C•l)

*Differential scanning calorimetry*

All protein samples were extensively dialyzed against 1x PBS, pH 7.4 prior to the DSC experiments. The experiments were done of a VP-DSC microcalorimeter. Samples at 19-22 µM concentration were scanned from 10 to 83°C with a scan rate of 90°C/hr with 4 sec averaging time and 5 min pre-scan thermostat. Data were analyzed using Origin software provided by the DSC manufacturer. 2-state or 3-state unfolding models were used, depending on the number of the observed DSC transitions

*Thermal Stability Assay (ThermoFluor Assay)*

A 384-well thermal stability assay using SYPRO orange was developed to determine the melting temperatures of purified proteins in APO (unbound) form and in the presence of ligand, adapted from Huynh *et al* (3). Mannoside compounds (methyl α-D-mannopyranoside (Sigma M6882) mimicking the natural ligand of FimH (mannose) were used to analyze association to the protein. FimH protein stock solutions were prepared by diluting proteins in 40 mM Tris pH 8, 400 mM NaCl (Assay Buffer) to 4 µM; SYPRO orange dye (Invitrogen S6650) was diluted 1:10 in Assay Buffer. 4 µM FimH mutants (5 µL) were mixed with 1:10 SYPRO orange dye (0.1µL) and either Assay Buffer or methyl α-D-mannopyranoside diluted in Assay Buffer (5 µL) (10 mM final concentration) for 10 µL final reaction volume in a MicroAmp EnduraPlate Optical 384-well plate (Applied Biosystems 4483285). The plate was subjected to melt curve analysis in a QuantStudio 5 Real-time PCR system (ThermoFisher) using a dissociation protocol from 20 ºC to 98 ºC, at 0.05 ºC / second. TAMRA was specified as the target and reporter, ROX as a passive reference. Data was plotted as a Maxwell–Boltzmann distribution and data analyzed as described previously (3) Melting temperature (T_m_) of the protein was calculated as the temperature at which half of the protein has dissociated; shift in melting temperature (ΔT_m_) was calculated by subtracting the T_m_ of the protein + ligand from the apo condition.

- 1. **X-ray crystallography of FimH-DSG TM**

For X-ray crystallography experiments, FimH-DSG TM was expressed in ExpiCHO cells (Thermo Fisher Scientific) as secreted proteins with C-terminal His tags. Cell culture supernatant was harvested and 1 M Tris pH 7.4 and 5 M NaCl were added to final concentrations of 20 mM and 150 mM respectively. A 5 kDa TFF cassette buffer was rinsed and equilibrated in 20 mM Tris pH 7.5 with 500 mM NaCl and 40 mM imidazole. Supernatant was concentrated 2-fold and diafiltered against 6 volumes of 20 mM Tris pH 7.5 500 mM NaCl 40 mM imidazole. Retentate was collected and filtered with a 0.2 µm bottletop filter. An XK26/20 column was packed with Ni-Sepharose 6 Fast Flow resin (Cytiva Life Sciences) and equilibrated with 5 column volumes of 20 mM Tris pH 7.5 500 mM NaCl 40 mM imidazole. Retentate was applied at half flow rate and washed until a stable baseline was reached (approximately 55 column volumes). Bound protein was eluted with 20 mM Tris, 500 mM NaCl, 500 mM imidazole, pH 7.5. Fractions containing the protein of interest were pooled and dialyzed in a 2 kDa dialysis cassette against 20 mM sodium acetate, pH 4.3 at 4 ºC with two buffer changes. Protein was applied to a SP-Sepharose cation exchange column (Cytiva Life Sciences) that had been equilibrated with the same buffer. Material bound to the cation-exchange resin was eluted with a linear gradient of NaCl using 20 mM sodium acetate, pH 4.3, 1 M NaCl buffer. Fractions were pooled, and dialyzed against TBS, pH 7.4.

Purified FimH-DSG TM was buffer exchanged against 20 mM Tris (pH 7.5) using PD-10 column and concentrated to 10 mg/ml. Crystallization was performed at 20 °C using sitting drop vapor diffusion method by mixing equal volumes of protein and reservoir solution containing 1 M sodium acetate (pH 4.5) and 25% (w/v) PEG3350. Crystals grew to their maximum size in ~ 7 days. Crystals were cryoprotected using the reservoir solution supplemented with 15% glycerol and flash frozen in liquid nitrogen. Diffraction data were collected at APS 17-ID. The data was processed using autoPROC (Global Phasing Limited) and the structure was solved using WT FimH-DSG complex (PDB code 4XOD) as a starting model (4)[.](#_ENREF_16) Model building and refinement were carried out using COOT [a](#_ENREF_51)nd BUSTER (Global Phasing Limited).

- 1. **Binning of monoclonal antibodies**

Of the 300 parents Mabs screened in the *E. coli* binding inhibition assay, 34 parents were inhibitory; hybridomas from these parents were expanded and 26/34 survived the expansion. From these, 30 ml supernatant was purified using protein A/G resin on gravity flow columns. 12 of the 26 parents had inhibitory activity, evaluated in a 7-point titration assay starting at 75 nM with 2-fold serial dilutions. These hybridomas were cloned, and 3 clones of each were screened again for neutralizing activity. This led to the selection of independent 10 Mab clones with neutralizing activity (**Table S7**).

Epitope binning and kinetics experiments were performed on an Octet HTX instrument. For each epitope binning experiment, Ni-NTA Biosensors (Sartorius Cat# 18-5103) were pre-wet in assay buffer containing 1x PBS 1% BSA 0.1 % Tween 20 for at least 10 minutes. To establish the initial baseline, Ni-NTA biosensors were immersed in assay buffer for 60 sec. His-tagged mammalian FimH_LD_ WT (mFimH_LD_ WT) was loaded at 5 µg/ml for 300 sec onto the baseline-established biosensors. mFimH_LD_ WT-loaded and baseline established biosensors were allowed to bind first Mab at 5 µg/ml for 5 min. the baseline was re-established in assay buffer for 3 min. Finally, the first Mab-loaded and baseline-established biosensors were allowed to bind competing Mab (5 µg / ml) for 300 seconds. The nm shift response was measured for all the antibodies under investigation. Antibodies competing against each other should have low binding response whereas the Mabs binding to different epitopes results in higher binding.

To measure kinetic measurements of different FimH Mabs, anti-mouse IgG Fc (AMC Biosensors, cat# 18-5090) were pre-wet in assay buffer for at least 10 min. The initial baseline was established with pre-wet AMC biosensors in assay buffer for 60 seconds. FimH monoclonal antibodies at 1 µg/ml were loaded onto baseline-established biosensors until the nm threshold reached 1 nm, or for 600 seconds. The baseline was re-established for Mab-loaded biosensors in assay buffer, for 3 minutes. Mab-loaded and baseline-established biosensors were allowed to bind 2-fold dilutions of m FimH_LD_ WT (starting at 100 nM), for 300 seconds followed by dissociation for 25 min. Results were processed in Data Evaluation v11.1 HT, by subtracting the sensorgram of 0 nM m FimH_LD_ WT blank from the rest of the dilutions. All sensorgrams were aligned to the baseline step followed by aligning the dissociation and association steps. Curves were fit with a Langmuir 1:1 global fit to obtain kinetic measurements. Average values obtained from 2 to 3 experiments were tabulated.

- 1. **Sample Preparation for FimH-DSG TM Fab complex Cryo-EM**

To prepare FimH-DSG TM complex with Fabs for analysis by cryo-EM, FimH-DSG TM was combined with a 1.25-fold molar excess each of ECO-329-2 Fab and ECO-445-3 Fab and incubated for 2 hours on ice. The mixture was fractionated by size-exclusion chromatography on a Superdex 200 5/150 GL gel filtration column pre-equilibrated in 20 mM HEPES pH 7.5, 150 mM NaCl. Fractions corresponding to the ternary complex were pooled and concentrated to 0.25 mg/ml in a 3 kDa MWCO Amicon centrifugal ultrafiltration concentrator. This sample was used for grid vitrification for cryo-EM.

Prior to vitrification, 0.3% β-octylglucoside was added to the sample. The sample was then subjected to centrifugation at 13,200 x *g* for 10 min to remove large aggregates. Gold Quantifoil R1.2/1.3 200 mesh grids were made hydrophilic by glow discharge in residual air at 15 mA for 30 seconds using a Pelco Easiglow. Grids were vitrified using a Vitrobot Mark IV at 4°C and 100% humidity. 4 μl of the sample supernatant was applied to Quantifoil Au 200 mesh R1.2/1.3 grids glow discharged in residual air, then blotted from both sides before plunge-freezing in liquid ethane. Grids were stored under liquid nitrogen until imaging.

- 1. **Cryo-EM Data Collection and Processing**

Grids for the FimH-DSG TM/ECO 329-2/ECO 445-3 complex were imaged in a Titan Krios G2 transmission electron microscope operated at 300 kV equipped with a Falcon 4i direct electron detector and Selectris X imaging filter. Screening and data collection were performed in EPU (Thermo Fisher Scientific). Movies in EER format were collected at 215,000x magnification (0.59 Å magnified pixel size at the specimen level) with a total electron dose of 50 e^-^/ Å^2^. A dataset of 7,584 movies was collected. All subsequent data processing was performed in CryoSPARC v3.3.1. Movies were subjected to patch motion correction (nominal pixel size = 0.59 Å, EER fractionation into 40 frames, without upsampling) and patch CTF correction. 348 manually picked particles were subjected to 2D classification to yield templates for template-based auto-picking, yielding 861,802 particles, which were extracted in 500-pixel (29.5 nm) boxes Fourier-cropped to 1.18 Å and subjected to multiple rounds of 2D classification. 199,906 particles were subjected to ab initio modeling in 4 classes. The most well-defined model, comprising 80,368 particles, was subjected to non-uniform 3D gold-standard refinement and reached a nominal resolution of 3.11 Å, according to the FSC=0.143 criterion. To improve the model quality, the constant regions of the Fabs and the FimH pilin domain were subtracted from the map in CryoSPARC, and the subtracted particles were subjected to 3D gold-standard local refinement against the model, using the pose/shift Gaussian prior during alignment and cross-validation-optimal non-uniform regularization. The resulting model was resolved to 3.12 Å with significantly improved quality.

Grids containing the FimH-DSG TM/ECO 440-2/ECO 454-3 complex were imaged in a Titan Krios G2 transmission electron microscope operated at 300 kV equipped with a Gatan K2 direct electron detector and Gatan Quantum LS imaging filter. Screening and data collection were performed in SerialEM (UC Boulder). Movies in TIFF format were collected at 165,000x (0.87 Å magnified pixel size at the specimen level) with a total electron dose of 50 e^-^/ Å^2^. A dataset of 6,498 movies was collected with the stage tilted by 30°. All subsequent data processing was performed using CryoSPARC v4.5.1. Movies were subjected to patch motion correction (nominal pixel size = 0.87 Å, EER fractionation into 40 frames, without upsampling) and patch CTF estimation. 1,767,438 particles were extracted in 360-pixel boxes after blob picking and subjected to multiple rounds of 2D classification to remove junk particles. A set of 214,141 particles was used as input for ab initio modeling in 3 classes. The best class, containing 78,393 particles, was used for gold-standard non-uniform refinement to yield a model at 4.77 Å resolution. This 3D model was used to generate 50 templates for autopicking. After template-based autopicking, 2,689,092 particles were extracted and subjected to 2D classification. The best 370,483 particles were combined with the original 214,141-particle set and subjected to 2D classification with removal of duplicate particles. 445,117 particles were selected for ab initio modeling in 4 classes, from which one class comprising 185,595 particles was selected for gold-standard non-uniform refinement, reaching 4.22 Å resolution. Further local gold-standard refinement using pose/shift Gaussian prior and a full solvent mask yielded a model at 4.24 Å resolution (FSC=0.143 criterion), but with significantly improved features. Local resolution for the Fab variable regions and the FimH lectin domain were sufficient to allow modeling.

- 1. **Model Building and Refinement**

To model the FimH-DSG TM complex with Fabs ECO 329-2 and ECO 445-3, atomic coordinates from the FimH DSG TM crystal structure reported in this manuscript and Fab chains from PDB entries 4U0R, 7C61, 6H3H, and 7DNH (identified by searching the heavy and light chain sequences of Fabs 329-2 and 445-3 against sequences available in the Protein Data Bank for the most similar structures) were rigid-body fitted into the cryo-EM map density. Sequence modifications were made to each of the Fab models to match the sequences of the Fabs used in the experiment, then the model was successively hand-refined into the map using Coot v0.9.8.1 in alternation with real-space refinement in Phenix v1.20 to produce the final model.

Modelling of the FimH-DSG TM complex with Fabs ECO 440-2 and ECO 454-3 began with rigid-body fitting of the FimH/329-2/445-3 model described above into the map, followed by deletion of the chains for Fab ECO 329-2. The sequences of the ECO 440-2 Fab heavy and light chains were used for a BLAST search against PDB sequences to identify the most similar Fabs with existing structures. PDB entry 3TT1, chain H was used for the heavy chain and PDB entry 1XGY, chain M was used for the light chain. Each chain was fitted as a rigid body into the map density for Fab ECO 440-2 and modified to match the correct 440-2 chain sequences. The model was rebuilt into the density by hand-building in Coot v0.9.8.1, alternating with real-space refinement in Phenix v1.20 to produce the final model.

The full cryo-EM data processing workflow and validation metrics can be found in the supplementary materials. Figures based on the structure were produced in PyMol v2.5.5, UCSF Chimera v1.16, and ChimeraX v1.4.

1. Supplementary data tables

Table A Bacterial binding inhibitory titers induced by immunization of mice with *E. coli* and mammalian produced FimH proteins

| Group | Responder rate (%) | # of responders (N=20) | Binding inhibition /geometric mean IC_50_ |
| --- | --- | --- | --- |
| Periplasmic FimH_LD_ WT | 55 | 11 | 300 |
| Periplasmic FimH_LD_ V27C L34C | 40 | 8 | 254 |
| Mammalian FimH_LD_ WT | 45 | 9 | 194 |
| Mammalian FimH_LD_ V27C L34C | 10 | 2 | 73 |
| Mammalian FimH-DSG WT | 75 | 15 | 529 |
| Mammalian FimH-DSG V27C L34C | 80 | 16 | 579 |
| Periplasmic FimCH | 67 | 12 (N=18) | 354 |

Table B Binding Kd of FimH mutants to octylbiphenylmannopyranoside ligand

| **Design category** | **FimH variant** | **Replicates** | **Average K_d_ / nM** |
| --- | --- | --- | --- |
| WT | FimH_LD_ WT | 12 | 0.2±0.0 |
|  | FimH_LD_ V27A | 3 | 0.2±0.0 |
| Reference mutations | FimH_LD_ R60P | 1 | 4.5 |
|  | FimH_LD_ V27A R60P | 6 | 34.6±8.2 |
|  | FimH_LD_ V27C L34C | 7 | 17.7±3.1 |
| Glycine switch mutations in the FimH_LD_ | FimH_LD_ G15P | 1 | >2000 |
|  | FimH_LD_ G15P V27A | 4 | >2000 |
|  | FimH_LD_ G15A | 1 | 46.7 |
|  | FimH_LD_ G15A V27A | 4 | >2000 |
|  | FimH_LD_ G16P | 1 | >2000 |
|  | FimH_LD_ G16P V27A | 4 | >2000 |
|  | FimH_LD_ G16A | 1 | 9.6 |
|  | FimH_LD_ G16A V27A | 3 | 30.2±0.8 |
| Glycine switch mutations in the FimH_LD_ | FimH_LD_ G15A G16A V27A | 4 | >2000 |
|  | FimH_LD_ V27A G65A | 1 | 4.6 |
| Cysteine pairs for disulfide bond stabilization in the FimH_LD_ | FimH_LD_ V28C N33C | 2 | 6.5±2.1 |
|  | FimH_LD_ V28C P157C | 1 | 7.7 |
|  | FimH_LD_ P26C V154C | 1 | 8.4 |
| Nonpolar-to-polar mutations in FimH_LD_ | FimH_LD_ L34T V27A | 1 | 0.4 |
|  | FimH_LD_ L34N V27A | 1 | 1.7 |
|  | FimH_LD_ A119T V27A | 1 | 0.4 |
|  | FimH_LD_ A119N V27A | 1 | 1 |
| Substitutions in the ligand binding site of FimH_LD_ | FimH_LD_ F1Y | 2 | 0.3 |
|  | FimH_LD_ F1W | 1 | 48.4 |
|  | FimH_LD_ F1M | 1 | >2000 |
|  | FimH_LD_ F1L | 1 | 534 |
|  | FimH_LD_ F1I | 1 | >2000 |
|  | FimH_LD_ F1V | 1 | 472 |
| Full length WT FimH | FimH-DSG WT | 12 | 23.4±7.6 |
|  | FimH-DSG V27A | 2 | 53.6±9 |
| Cysteine pairs for disulfide bond stabilization in the FimH_LD_, in full length FimH | FimH-DSG V27C L34C | 3 | 59.9±28.2 |
| Glycine switch mutations in the FimH_LD_, in full length FimH | FimH-DSG G15A V27A | 2 | >2000 |
|  | FimH-DSG G16A V27A | 2 | >2000 |
|  | FimH-DSG G15A G16A V27A | 6 | >2000 |
| Cavity-filling mutations at the Pilin-Lectin interface of FimH-DSG | FimH-DSG A115I | 1 | 9.8 |
|  | FimH-DSG V185I | 1 | 21 |

Table C Melting temperature of FimH mutants in apo state and in the presence of methyl alpha-D-mannopyranoside

| **Design category** | **FimH variant** | **Replicates** | **Tm (Average) / ºC** | **StdDev of Tm / ºC** | **ΔTm (Average) / ºC** | **StdDev of ΔTm / ºC** |  |
| --- | --- | --- | --- | --- | --- | --- | --- |
| WT | FimH_LD_ WT | 14 | 61.5 | 0.8 | 10.8 | 0.8 | |
|  | FimH_LD_ V27A | 6 | 59.9 | 0.9 | 10.0 | 0.6 | |
| Reference | FimH_LD_ R60P | 3 | 57.5 | 2.2 | 7.9 | 1.5 | |
|  | FimH_LD_ V27A R60P | 3 | 60.1 | 0.4 | 3.1 | 0.4 | |
| Glycine switch mutations in the FimH_LD_ | FimH_LD_ G15A | 4 | 56.0 | 0.4 | 3.8 | 0.3 | |
|  | FimH_LD_ G15P | 3 | 56.9 | 1.7 | 2.0 | 1.4 | |
|  | FimH_LD_ G16A | 3 | 55.7 | 2.0 | 4.7 | 2.3 | |
|  | FimH_LD_ G16P | 2 | 55.3 | 0.6 | 1.5 | 0.3 | |
|  | FimH_LD_ G15A V27A | 4 | 57.7 | 0.5 | 1.7 | 0.2 | |
|  | FimH_LD_ G15P V27A | 3 | 58.3 | 0.4 | 0.9 | 0.7 | |
|  | FimH_LD_ G16A V27A | 2 | 57.3 | 0.7 | 2.4 | 1.2 | |
|  | FimH_LD_ G16P V27A | 2 | 58.2 | 0.6 | 0.6 | 0.6 | |
|  | FimH_LD_ G15A G16A V27A | 4 | 58.9 | 0.7 | 0.5 | 0.6 | |
| Cysteine pairs for disulfide bond stabilization in the FimH_LD_ | FimH_LD_ V28C N33C | 4 | 65.5 | 1.1 | 2.8 | 0.2 | |
|  | FimH_LD_ V27C L34C | 5 | 51.5 | 0.9 | 7.3 | 0.5 | |
|  | FimH_LD_ P26C V154C | 3 | 59.7 | 1.4 | 6.1 | 0.9 | |
|  | FimH_LD_ P26C V156C | 1 | 58.9 | NA | 5.4 | NA | |
|  | FimH_LD_ Q32C Y108C | 1 | 61.1 | NA | 9.8 | NA | |
|  | FimH_LD_ P26C V154C | 2 | 58.9 | 0.3 | 6.6 | 0.5 | |
|  | FimH_LD_ S62C T86C | 1 | 58.3 | NA | 11.7 | NA | |
|  | FimH_LD_ S62C L129C | 1 | 57.2 | NA | 12.7 | NA | |
|  | FimH_LD_ Y64C A127C | 1 | 60.2 | NA | 12.9 | NA | |
|  | FimH_LD_ V112C T158C | 1 | 59.6 | NA | 15.2 | NA | |
|  | FimH_LD_ V118C V156C | 1 | 59.4 | NA | 10.1 | NA | |
|  | FimH_LD_ P12C A18C | 1 | 51.4 | NA | 7.8 | NA | |
|  | FimH_LD_ G14C F144C | 1 | 49.2 | NA | -0.1 | NA | |
|  | FimH_LD_ L68C F71C | 1 | 49.9 | NA | 12.2 | NA | |
|  | FimH_LD_ S113C G116C | 1 | 59.8 | NA | 9.3 | NA | |
|  | FimH_LD_ A119C V155C | 1 | 59.1 | NA | 14.5 | NA | |
| Substitutions in the ligand binding site of FimH_LD_ | FimH_LD_ F1I | 2 | 55.2 | 0.0 | 0.3 | 1.2 | |
|  | FimH_LD_ F1L | 2 | 60.1 | 0.2 | 0.8 | 0.6 | |
|  | FimH_LD_ F1M | 3 | 52.7 | 1.5 | 1.1 | 1.6 | |
|  | FimH_LD_ F1V | 3 | 45.8 | 11.6 | 7.4 | 11.5 | |
|  | FimH_LD_ F1W | 3 | 52.9 | 0.6 | 4.5 | 0.6 | |
|  | FimH_LD_ F1Y | 3 | 54.2 | 0.2 | 10.1 | 0.3 | |
| Nonpolar-to-polar mutations in FimH_LD_ | FimH_LD_ L34S V27A | 1 | 52.0 | NA | 9.9 | NA | |
|  | FimH_LD_ L34T V27A | 1 | 53.2 | NA | 10.5 | NA | |
|  | FimH_LD_ L34N V27A | 1 | 47.3 | NA | 13.1 | NA | |
|  | FimH_LD_ A119S V27A | 1 | 59.8 | NA | 8.5 | NA | |
|  | FimH_LD_ A119T V27A | 1 | 59.5 | NA | 9.2 | NA | |
|  | FimH_LD_ A119N V27A | 1 | 57.9 | NA | 7.6 | NA | |
|  | FimH_LD_ V27A G65A | 1 | 60.6 | NA | 6.2 | NA | |
| Full length WT FimH | FimH-DSG WT | 11 | 71.3 | 1.0 | 2.2 | 0.4 | |
| Cysteine pairs for disulfide bond stabilization in the FimH_LD_, in full length FimH | FimH-DSG V27C L34C | 7 | 64.1 | 2.2 | 1.1 | 0.7 | |
| Full length WT FimH | FimH-DSG V27A | 5 | 72.6 | 0.6 | -0.3 | 0.2 | |
| Glycine switch mutations in the FimH_LD_, in full length FimH | FimH-DSG G15A V27A | 5 | 73.0 | 0.6 | -0.1 | 0.3 | |
|  | FimH-DSG G16A V27A | 5 | 72.3 | 0.5 | 0.0 | 0.1 | |
|  | FimH-DSG G15A G16A V27A | 7 | 73.2 | 0.7 | 0.0 | 0.1 | |
| Cavity-filling mutations at the Pilin-Lectin interface of FimH-DSG | FimH-DSG A115I | 1 | 68.5 | NA | 4.1 | NA | |
|  | FimH-DSG V185I | 2 | 71.4 | 0.5 | 2.5 | 0.4 | |
|  | FimH-DSG DSG V3I | 1 | 70.7 | NA | 3.1 | NA | |
|  | FimH-DSG V163I | 1 | 70.4 | NA | 3.5 | NA | |
| Ligand binding blocking mutation in FimH_LD_, in full length FimH | FimH-DSG Q133K | 1 | 71.6 | NA | 1.7 | NA | |
|  | FimH-DSG V27A Q133K | 1 | 75.1 | NA | 0.1 | NA | |
| Ligand binding blocking mutation in FimH_LD_, in full length FimH | FimH-DSG G15A G16A V27A Q133K | 1 | 73.9 | NA | 1.3 | NA | |

***NA = not applicable***

Table D Geometric mean bacterial binding inhibitory titers induced by FimH_LD_ and FimH-DSG mutants proteins in mice

| **Protein** | **IC_50_ GMTs** | | **Responder rate (%)** | | **Responders (n)** | | **Mice (n)** | |
| --- | --- | --- | --- | --- | --- | --- | --- | --- |
|  | **PD2** | **PD3** | **PD2** | **PD3** | **PD2** | **PD3** | **PD2** | **PD3** |
| FimH_LD_ WT | 89 | 191 | 20 | 40 | 4 | 8 | 20 | 20 |
| FimH_LD_ V27A | 104 | 439 | 26 | 61 | 5 | 11 | 19 | 18 |
| FimH-DSG V27A | 1175 | 6102 | 78 | 100 | 14 | 18 | 18 | 18 |
| FimH_LD_ G15A V27A | 57 | 683 | 5 | 53 | 1 | 10 | 20 | 19 |
| FimH-DSG G15A V27A | 1740 | 3400 | 84 | 100 | 16 | 19 | 19 | 19 |
| FimH_LD_ G15P V27A | 58 | 346 | 5 | 42 | 1 | 8 | 20 | 19 |
| FimH_LD_ G16A V27A | 93 | 1193 | 13 | 69 | 2 | 11 | 16 | 16 |
| FimH_LD_ G16P V27A | 94 | 352 | 10 | 45 | 2 | 9 | 20 | 20 |
| FimH_LD_ G15A G16A V27A | 111 | 1307 | 26 | 63 | 5 | 12 | 19 | 19 |
| FimH-DSG G15A G16A V27A | 1869 | 2386 | 84 | 95 | 16 | 18 | 19 | 19 |
| FimH_LD_ V27A R60P | 212 | 1056 | 32 | 63 | 6 | 12 | 19 | 19 |

Table E Neutralizing epitopes are preserved in four aglycosylated variants of the FimH DsG triple mutant antigen

|  | **Response (nm)** | | | | |
| --- | --- | --- | --- | --- | --- |
|  | **Aglycosylated FimH-DSG TM variants** | | | | **Glycosylated FimH-DSG TM** |
| **mAb** | N228S N235S  N=1 | T230A T237A  N=1 | N228G N235G  N=1 | N228Q N235Q  N=1 | N235  N=1 |
| **299-3** | 0.22 | 0.23 | 0.22 | 0.22 | 0.25 |
| **306-2** | 0.24 | 0.26 | 0.25 | 0.26 | 0.27 |
| **926** | 0.49 | 0.47 | 0.48 | 0.49 | 0.55 |
| **329-2** | 0.24 | 0.23 | 0.25 | 0.24 | 0.27 |
| **440-2** | 0.13 | 0.11 | 0.11 | 0.13 | 0.17 |
| **445-3** | 0.27 | 0.28 | 0.31 | 0.27 | 0.30 |

Table F X-ray data collection and refinement statistics for FimH DSG

|  | **FimH DSG (pdb code: 8V3J)** |
| --- | --- |
| **Data collection** |  |
| Space group | C121 |
| Cell dimensions |  |
| *a*, *b*, *c* (Å) | 140.04, 149.26, 99.35 |
| α, β, g (^o^) | 90.00, 130.03, 90.00 |
| Resolution (Å) | 87.09-1.90 (2.04-1.90)* |
| *R*_sym_ or *R*_merge_ | 0.05 (0.64) |
| *I* / s*I* | 13.5 (1.33) |
| Completeness (%) | 70.4 (49.2) |
| Redundancy | 3.3 (2.3) |
|  |  |
| **Refinement** |  |
| Resolution (Å) | 87.09-1.90 |
| No. reflections | 86513 |
| *R*_work_ / *R*_free_ | 0.207 / 0.230 |
| No. atoms |  |
| Protein | 8054 |
| Water | 857 |
| *B*-factors (Å^2^) |  |
| Mean, all atoms | 41.0 |
| R.m.s. deviations |  |
| Bond lengths (Å) | 0.008 |
| Bond angles (°) | 0.99 |
| **Ramachandran plot**  Favored (%) | 99.0 |
| Allowed (%) | 1.0 |
| Disallowed (%) | 0 |

*Values in parentheses are for highest-resolution shell.

Table G Identification of novel FimH inhibitory antibodies

Mabs against FimH were screened for ability to inhibit bacterial binding to ligand in the bacterial inhibition assay. Resulting Mabs were subjected to a series of competition experiments (described in Supplementary materials) to identify groups of antibodies likely binding similar epitopes which led to the identification of 4 bins (bin 1 including antibodies that bound to the ligand binding site, all others bound outside of the ligand binding site). This table shows inhibitory titers of one antibody representing each bin and its relative FimH binding affinity. Two Mabs, 440-2 and 445-3, were derived from mice immunized with a conformationally locked version of FimH, FimH-DSG LM (V27C L34C).

| **Site** | **Mab specificity** | **FimH Mabs** | **Inhibitory (IC_50_) titer, µg/ml** | **Kinetic Measurements with FimH-DSG WT (Averages with Stdev)** | | | | |
| --- | --- | --- | --- | --- | --- | --- | --- | --- |
|  |  |  |  | **k_a_ (1/Ms)** | **k_dis_ (1/s)** | **K_D_ (nM)** | **Replicates** | **Elicited antigen** |
| 1 | Ligand binding site epitope (reference monoclonal antibodies) | 475 | 26.4 | (1.9±0.26) x10^5^ | (5.7±1.8) x10^-4^ | 3.1 | 8 |  |
|  |  | 926 | 0.4 | (7.1±0.53) x10^5^ | (6.5±0.48) x10^-4^ | 0.9 | 5 |  |
| 1 | Ligand binding site epitope | 299-3 | 1.4 | (4.1±0.25) x10^5^ | (3.4±0.33) x10^-4^ | 0.8 | 4 | FimH-DSG WT |
|  |  | 304-1 | 0.3 | (4.7±0.43) x10^5^ | (1.0±0.16) x10^-4^ | 0.2 | 7 |  |
|  |  | 306-2 | 4.9 | (7.5±0.30) x10^5^ | (4.8±0.15) x10^-4^ | 0.6 | 5 |  |
|  |  | 313-1 | 11.9 | (2.6±0.23) x10^5^ | (5.3±0.12) x10^-4^ | 2.1 | 5 |  |
|  |  | 330-2 | 14.3 | (5.9±0.81) x10^5^ | (1.1±0.16) x10^-4^ | 1.9 | 5 |  |
|  |  | 338-4 | 2.5 | (1.5±0.097) x10^5^ | (2.1±0.14) x10^-4^ | 1.4 | 5 |  |
| 2 | Non-ligand binding site epitope | 327-3 | 1.0 | (5.8±0.15) x10^5^ | (8.0±0.63) x10^-4^ | 0.1 | 5 |  |
|  |  | 329-2 | 3.3 | (4.7±0.35) x10^5^ | (6.7±0.18) x10^-4^ | 0.1 | 5 |  |
| 3 | Non-ligand binding site epitope | 445-3 | 1.3 | (5.8±0.42) x10^5^ | (7.5±0.28) x10^4^ | 0.1 | 5 | FimH DSG-LM |
| 4 | Non-ligand binding site epitope | 440-2 | 94.8 | (9.4±1.9) x10^4^ | (6.2±0.82) x10^-4^ | 7.0 | 5 |  |

Table H Mab binding to FimH_LD_ mutants

| **FimH variant** | **Response (nm)** | | |
| --- | --- | --- | --- |
|  | **Monoclonal antibody** | | |
|  | **299-3** | **304-1** | **440-2** |
| FimH_LD_ WT | 3.10 | 3.02 | 0.04 |
| FimH_LD_ V27A | 0.44 | 0.54 | 0.06 |
| FimH_LD_ V27A R60P | 3.19 | 3.03 | 0.85 |
| FimH_LD_ V27A G15A | 3.31 | 3.03 | 0.78 |
| FimH_LD_ V27A G15P | 3.07 | 2.95 | 0.62 |
| FimH_LD_ V27A G16A | 3.44 | 3.20 | 0.75 |
| FimH_LD_ V27A G16P | 3.26 | 3.16 | 0.88 |
| FimH_LD_ G15A G16A V27A | 3.34 | 3.06 | 0.79 |
| FimH_LD_ V27C L34C | 3.11 | 2.77 | 0.81 |
| FimH_LD_ V28C N33C | 3.054 | 2.74 | 0.72 |
| FimH_LD_ P26C V154C | 3.11 | 2.89 | 0.83 |

Table I Mab binding to FimH-DSG variants

| **FimH variant** | **Response (nm)** | | |
| --- | --- | --- | --- |
|  | **Monoclonal antibody** | | |
|  | **299-3** | **304-1** | **440-2** |
| FimH-DSG WT | 2.44 | 2.22 | 0.38 |
| FimH-DSG V27A | 2.48 | 2.17 | 0.35 |
| FimH-DSG V27C L34C | 2.35 | 2.23 | 0.33 |
| FimH-DSG V27A G15A | 2.52 | 2.30 | 0.37 |
| FimH-DSG V27A G16A | 2.69 | 2.42 | 0.42 |
| FimH-DSG G15A G16A V27A | 2.68 | 2.30 | 0.49 |

Table J Cryo-EM data and refinement statistics

|  | **FimH DSG TM + Fab 440-2 + Fab 454-3**  **(EMD-46596)**  **(PDB 9D6F)** | **FimH DSG TM + Fab 329-2 + Fab 454-3**  **(EMD-43048)**  **(PDB 8V93)** |
| --- | --- | --- |
| **Data collection and processing** |  |  |
| Magnification | 165,000x | 215,000x |
| Voltage (kV) | 300 | 300 |
| Electron exposure (e–/Å^2^) | 50 | 50 |
| Defocus range (μm) | -3.2 to -1.2 | -2.4 to -0.6 |
| Pixel size (Å) | 0.87 | 0.59 |
| Symmetry imposed | C1 | C1 |
| Initial particle images (no.) | 2,689,092 | 861,802 |
| Final particle images (no.) | 185,595 | 80,368 |
| Map resolution (Å)  FSC threshold | 4.24  (0.143) | 3.12  (0.143) |
|  |  |  |
| **Refinement** |  |  |
| Initial model used (PDB code) | 3TT1, 1XGY | 4U0R, 7C61, 6H3H, 7DNH |
| Model resolution (Å)  FSC threshold | 4.4  0.5 | 3.9  0.5 |
| Map sharpening *B* factor (Å^2^) | 201.6 | 82.7 |
| Model composition  Non-hydrogen atoms  Protein residues  Ligands | 4,672  610  0 | 4,677  613  0 |
| *B* factors (Å^2^)  Protein  Ligand | 52.85/177.94/113.91  N/A | 29.58/100.90/56.49  N/A |
| R.m.s. deviations  Bond lengths (Å)  Bond angles (°) | 0.004  0.927 | 0.005  0.969 |
| Validation  MolProbity score  Clashscore  Poor rotamers (%) | 1.74  4.59  0 | 1.82  6.54  0 |
| Ramachandran plot  Favored (%)  Allowed (%)  Disallowed (%) | 91.41  8.25  0.34 | 92.82  7.01  0.17 |

1. **Supplementary figures**

**Figures: can be found in Silmon de Monerri et al supplementary figures.pdf**

**Figure legends**

Fig A Confirmation of correct signal peptide cleavage of mammalian expressed FimH_LD_ .

(A) Upper panel, extracted ion Chromatogram (XIC) of both the expected correctly processed tryptic peptide (FACK) and the peptide found (WSFACK) for FimH_LD_ WT produced in mammalian cells using the native FimH signal peptide. The signals in both chromatograms were normalized to each other; the data suggests the protein with WT leader was processed incorrectly. Lower panels, extracted ion Chromatogram (XIC) of both the expected incorrectly processed tryptic peptide (TGFACK) and the peptide found (FACK) for FimH_LD_ WT produced in mammalian cells using the mouse IgGκ signal peptide. The signals in both chromatograms were normalized to each other; the data suggests the protein was processed correctly. Sequences of the native FimH and mouse IgGκ signal peptides are highlighted in red. (B). MS/MS spectra showing the evidence for the assignment of the FACK peptide demonstrating the correct processing of the mouse IgK signal peptide. (C) MS/MS spectra showing the evidence for the assignment of the WSFACK peptide demonstrating the incorrect processing of the native FimH signal peptide.

**Fig B Optimization of full length FimH design and glycosylation**

**(A)** Expi293 cells were transfected with constructs encoding FimH-DSG with linkers (DNKQ, GGSSGG, etc.) and the FimCH complex. Raw culture supernatants collected 96 hours post transfection were loaded on an SDS-PAGE gel and stained with Coomassie blue. **(B)** Deconvoluted spectra of the intact protein demonstrating the complexity of the glycosylation of the FimH-DSG TM protein. **(C)** Deconvoluted spectra of the intact FimH-DSG TM protein after deglycosylation using PNGase F. Note, the resulting spectra is in good agreement with theoretical when considering disulfide bonds.

Fig C Informative regions of CD spectra of FimH_LD_ and FimH-DSG mutants

CD spectra in the far-UV **(A)**, reporting on the secondary structure, and near-UV **(B)**, reporting on the tertiary structure of FimH variants.

Fig D FimH-DSG TM aglycosylation variants have similar ability to elicit antibodies that inhibit bacterial binding compared to glycosylated parent

**(A)** Groups of 20 CD-1 mice (7-9 weeks old) were vaccinated subcutaneously with 0.1 mL of 10 µg of FimH-DSG TM antigens adjuvanted with 20 µg QS21/PS80 at weeks 0, 4 and 8. **(B)** Sera from week 6 and week 10 timepoints were tested for activity in the *E. coli* binding inhibition assay with geometric mean IC_50_ titers reported. Inhibitory titers were determined from serial dilution of sera from vaccinated mice and represent the reciprocal of the dilution of serum at which 50% of bacteria remain bound to the yeast mannan-coated assay plate and are shown for post dose 3 timepoint. Statistical significance (p-value) of differences in responses between groups was determined using an unpaired t-test with Welch’s correction applied to log-transformed data. Proportion of animals in each group responding to vaccine by exhibiting measurable IC_50_ titers are reported as % responder rates. **(C)** Figure displaying individual IC_50_ values; bars represent geometric mean and 95% confidence interval.

**Fig E FimH-DSG TM can be purified to homogeneity from ExpiCHO supernatants**

Purification profiles and analysis of FimH-DSG WT (left panels) and FimH-DSG TM (right panels). **(A)** Elution profile on SP-Sepharose column. **(B)** SDS-PAGE analysis of eluted fractions. **(C)** Analytical SEC profile. **(D)** Normalized amounts of monosaccharides (µg / mg protein) detected by HPAEC-PAD in various SP-Sepharose fractions from FimH-DSG WT (left) and the main peak of FimH-DSG TM (right).

**Fig F Visualization of linker region in structure of FimH-DSG TM**

Electron density of the glycine-serine linker region. FimH_LD_ is shown in orange, FimH_PD_ is shown in grey, the donor-strand peptide in pink and the glycine-serine linker in green.

Fig G Ability of Octylmannopyranoside to interfere with binding of monoclonal antibodies to FimH_LD_ WT

FimH_LD_ WT was incubated with a two-fold dilution of Ocytlmannopyranoside ligand before loading onto Octet Ni-NTA biosensors. FimH Mabs were allowed to bind at 5 µg/ml to the pre-bound FimH_LD_ WT and nanometer response measured for each dilution of the ligand. Mabs 327-3, 445-3 and 329-2 do not directly interfere with ligand binding.

Fig H Cryo-EM processing pipeline for FimH-DSG TM /329-2/445-3

Cryo-EM data processing workflow in CryoSPARC for the FimH-DSG TM/329-2/445-3 ternary complex, with final map colored by local resolution and Fourier shell correlation curves from gold-standard refinement.

Fig I Cryo-EM processing pipeline – FimH-DSG TM/440-2/445-3

Cryo-EM data processing workflow in Relion for the FimH-DSG TM/440-2/445-3 ternary complex.

**References**

1. Chorro L, Li Z, Chu L, Singh S, Gu J, Kim JH, et al. Preclinical Immunogenicity and Efficacy of Optimized O25b O-Antigen Glycoconjugates To Prevent MDR ST131 E. coli Infections. Infect Immun. 2022;90(4):e0002222.

2. Rabbani S, Fiege B, Eris D, Silbermann M, Jakob RP, Navarra G, et al. Conformational switch of the bacterial adhesin FimH in the absence of the regulatory domain: Engineering a minimalistic allosteric system. J Biol Chem. 2018;293(5):1835-49.

3. Huynh K, Partch CL. Analysis of protein stability and ligand interactions by thermal shift assay. Curr Protoc Protein Sci. 2015;79:28 9 1- 9 14.

4. Sauer MM, Jakob RP, Eras J, Baday S, Eris D, Navarra G, et al. Catch-bond mechanism of the bacterial adhesin FimH. Nat Commun. 2016;7:10738.
