# Supplementary figures and images for: Structure-based design of an immunogenic, conformationally stabilized FimH antigen for a urinary tract infection vaccine
Source: PLoS Pathog. 2025 Feb 19;21(2):e1012325. doi: 10.1371/journal.ppat.1012325 (PMC12136410; doi:10.1371/journal.ppat.1012325)

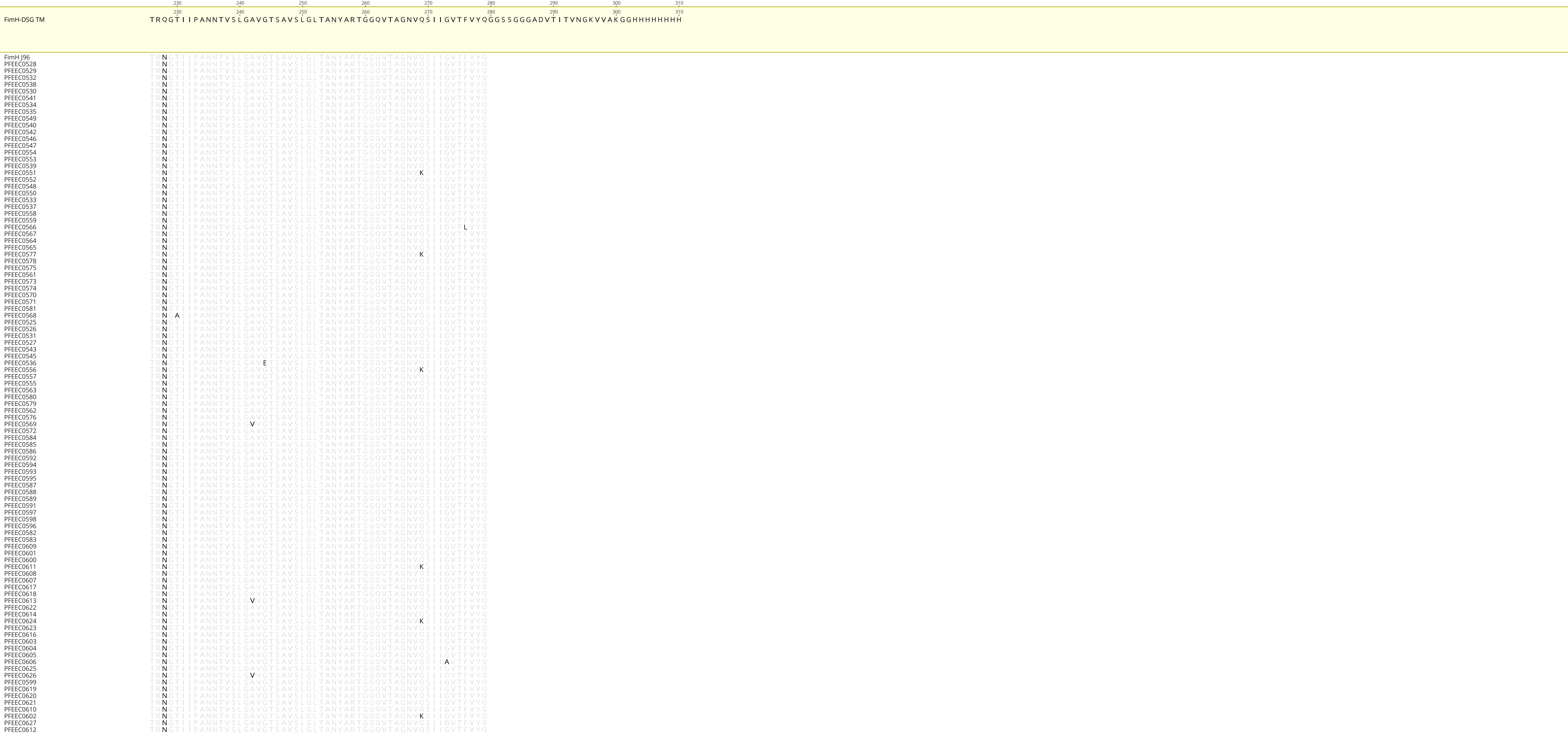

Supplement: S2 File — Isolates were obtained from the Antimicrobial Testing Leadership and Surveillance (ATLAS) database). 102 FimH sequences from UTI isolates are aligned against FimH-DSG TM and full length FimH from strain J96 (set as the reference sequence). Residues that differ from the reference FimH sequence from J96 strain are shown in black. Residues mapped to the epitopes of Mabs 329-2 (pink), 440-2 (green), 445-3 (blue) derived from cryoEM analysis are shown along with epitopes of previously identified Mabs 824 (orange), 926 (red) and 475 (turquoise). Image created using Geneious version 2023.0 created by Biomatters. Available from https://www.geneious.com. (PDF) [file ppat.1012325.s005.pdf]
