# Supplementary material for: Structure-based design of an immunogenic, conformationally stabilized FimH antigen for a urinary tract infection vaccine
Source: PLoS Pathog. 2025 Feb 19;21(2):e1012325. doi: 10.1371/journal.ppat.1012325 (PMC12136410; doi:10.1371/journal.ppat.1012325)
Supplement: S1 File — FimH-DSG variants wherein different lengths of Glycine-Serine linker were tested are labelled e.g. FimH-DSG 7 GS (denoting FimH-DSG with a 7 amino acid Glycine-Serine linker) and so on. Residues that differ from the reference FimH sequence from J96 strain are shown in black. Image created using Geneious version 2023.0 created by Biomatters. Available from https://www.geneious.com. (PDF) [file ppat.1012325.s004.pdf]

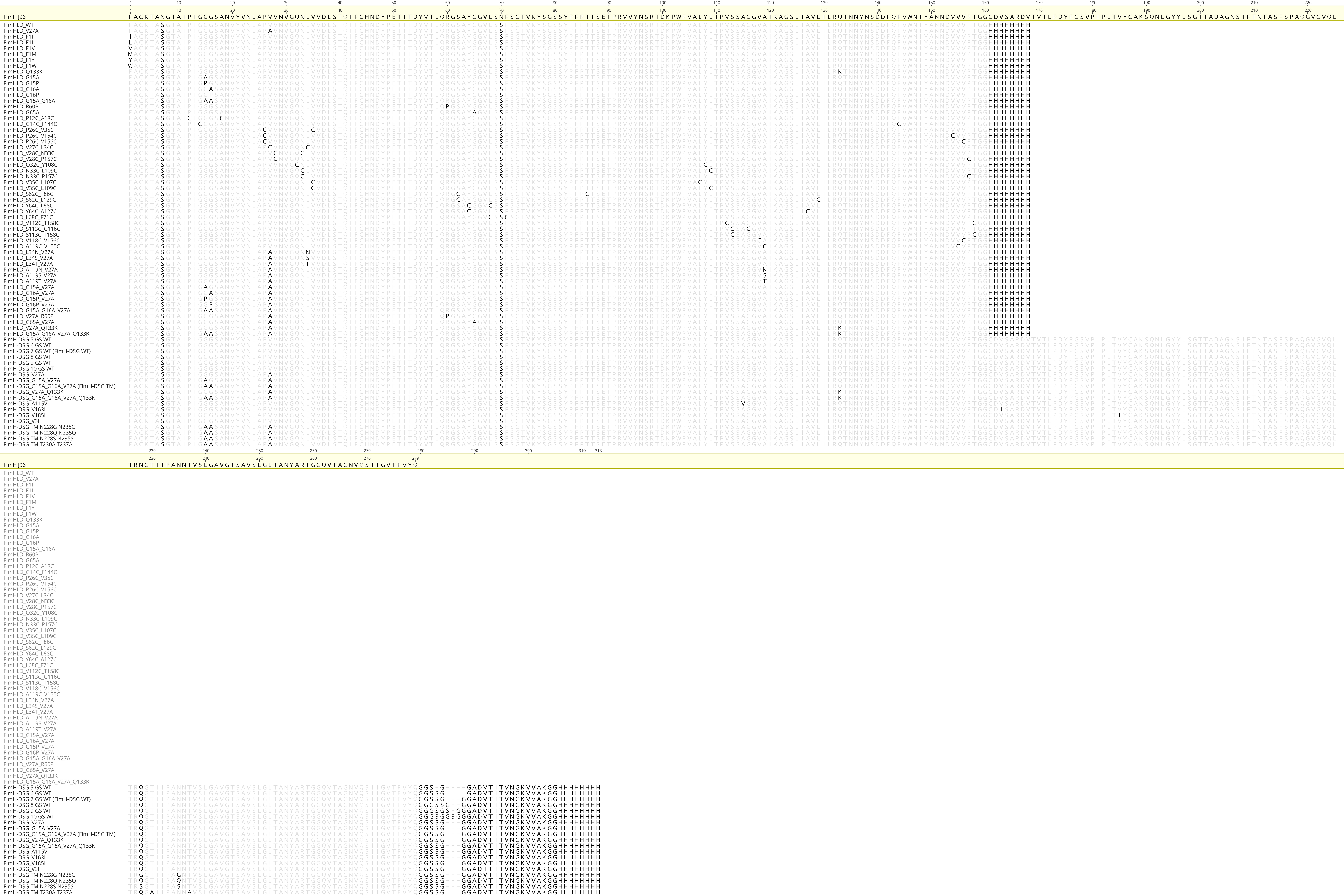

FimH J96
FACKTANGTAIPIGGGSANVYVNLAPVVNVGQNLVVDLSTQIFCHNDYPETITDYVTLQRGSAYGGVLSNFSGTVKYSGSSYPFPTTSETPRVVYNSRTDKPWPVALYLTPVSSAGGVAIKAGSLIAVLILRQTNNYNSDDFQFVWNIYANNDVVVPTGGCDVSARDVTVTLPDYPGSVPIPLTVYCAKSQNLGYYLSGTTADAGNSIFTNTASFSPAQGVGVQL
TRNGTIIPANNTVSLGAVGTSAVSLGLTANYARTGGQVTAGNVQSIIGVTFVYQ
FimHLD_WT
FimHLD_V27A
FimHLD_F1I
FimHLD_F1L
FimHLD_F1V
FimHLD_F1M
FimHLD_F1Y
FimHLD_F1W
FimHLD_Q133K
FimHLD_G15A
FimHLD_G15P
FimHLD_G16A
FimHLD_G16P
FimHLD_G15A_G16A
FimHLD_R60P
FimHLD_G65A
FimHLD_P12C_A18C
FimHLD_G14C_F144C
FimHLD_P26C_V35C
FimHLD_P26C_V154C
FimHLD_P26C_V156C
FimHLD_V27C_L34C
FimHLD_V28C_N33C
FimHLD_V28C_P157C
FimHLD_Q32C_Y108C
FimHLD_N33C_L109C
FimHLD_N33C_P157C
FimHLD_V35C_L107C
FimHLD_V35C_L109C
FimHLD_S62C_T86C
FimHLD_S62C_L129C
FimHLD_Y64C_L68C
FimHLD_Y64C_A127C
FimHLD_L68C_F71C
FimHLD_V112C_T158C
FimHLD_S113C_G116C
FimHLD_S113C_T158C
FimHLD_V118C_V156C
FimHLD_A119C_V155C
FimHLD_L34N_V27A
FimHLD_L34S_V27A
FimHLD_L34T_V27A
FimHLD_A119N_V27A
FimHLD_A119S_V27A
FimHLD_A119T_V27A
FimHLD_G15A_V27A
FimHLD_G16A_V27A
FimHLD_G15P_V27A
FimHLD_G16P_V27A
FimHLD_G15A_G16A_V27A
FimHLD_V27A_R60P
FimHLD_G65A_V27A
FimHLD_V27A_Q133K
FimHLD_G15A_G16A_V27A_Q133K
FimH-DSG 5 GS WT
FimH-DSG 6 GS WT
FimH-DSG 7 GS WT (FimH-DSG WT)
FimH-DSG 8 GS WT
FimH-DSG 9 GS WT
FimH-DSG 10 GS WT
FimH-DSG_V27A
FimH-DSG_G15A_V27A
FimH-DSG_G15A_G16A_V27A (FimH-DSG TM)
FimH-DSG_V27A_Q133K
FimH-DSG_G15A_G16A_V27A_Q133K
FimH-DSG_A115V
FimH-DSG_V163I
FimH-DSG_V185I
FimH-DSG_V3I
FimH-DSG TM N228G N235G
FimH-DSG TM N228Q N235Q
FimH-DSG TM N228S N235S
FimH-DSG TM T230A T237A
